# Mindfulness and Executive Functions: Making the Case for Elementary School Practice

**DOI:** 10.3390/ejihpe10010039

**Published:** 2020-03-04

**Authors:** Anne Ritter, Isabel Alvarez

**Affiliations:** Department of Social and Systematic Pedagogy Autonomous, University of Barcelona, 08193 Bellaterra, Cerdanyola del Vallès, Barcelona, Spain; anne.ritter@e-campus.uab.cat

**Keywords:** Mind Yeti, school-based intervention, inhibition, cognitive flexibility, working memory

## Abstract

This study explores the use of mindfulness school-based intervention program in an elementary school. Mindfulness training is an accepted and effective didactic approach to improve the executive functions (EFs) of elementary school students. This study aimed to evaluate the effects of the Mind Yeti program on the executive functions of elementary school students. A diverse sample of third, fourth, and fifth grade elementary school students (*n* = 177) participated in their natural classroom setting, with six sessions per week for six weeks. Students self-reported their EFs on the Executive Function Student Questionnaire (EFSQ) pre- and posttest. Paired-sample t-tests indicated that students significantly improved on the three of the six EFs examined. Additionally, students in fifth grade responded better to Mind Yeti than students in third grade. Results were consistent with the hypothesis, suggesting that Mind Yeti is an appropriate and effective intervention for improving the EFs of students.

## 1. Background

The executive functions (EFs) are a set of interrelated, neurologically based behavioral and metacognitive skills that set the foundation for personal and academic success. These skills are extremely important for the completion of academic tasks as they allow students to control their emotions, initiate tasks, manage time, pay attention, and remember details [1,2]. Students who have problems with EFs (i.e., executive dysregulation), may have poor follow-through on assignments, display disorganization, emotional outbursts, and difficulty with language and comprehension [2]. Additionally, students with executive dysregulation may have challenges controlling their behaviors and emotions, which could cause them to experience social rejection and bullying [3]. The adverse effects of executive dysregulation may continue into adulthood, “negatively affecting academic achievement, relationships, and employment” [4]. Therefore, it is imperative to provide students with tools and strategies to strengthen their EFs, especially those in the upper elementary grades who are increasingly required to complete more complex tasks and are often socially fragile.

One tool that has been gaining momentum and has shown promising findings in improving the EFs of school children is mindfulness training [5]. “According to Kabat-Zinn (1990), “mindfulness means paying attention in a particular way: on purpose, in the present moment, and nonjudgmentally [6].” Students who engaged in mindfulness training have shown improvements in attention, self-regulation, and mental processing [4], as well as cognition, academic performance, behavior, and EFs [7]. Students who meditate display “higher levels of mindfulness, better attentional performance, and higher cognitive flexibility” [8]. Additionally, they are more likely to substitute automatic responses with ones that are deliberate and flexible in changing situations [9]. Mindfulness training can elicit structural changes in the brain, which is thought to improve EFs as it “fosters enhanced resilience and more optimal brain function” [10].

With regard to the use of instruments for assessing mindfulness with young children there are remarkable studies worth to mention like the Child Observation of Mindfulness Measure (C-OMM) [11] proving to be relevant during teacher-directed activities only or the Individualized Classroom Assessment Scoring System (In-Class) to assess children’s behaviors as they pertain to desirable social behaviors requiring trained observers to note, interpret, and code select children’s behaviors related to their experiences with teachers, peers and tasks [12].

In addition, there are multiple MBI programs and curricula marketed to schools for use with elementary school students and they have “differences in the underlying content, methods, dosage, and effectiveness” [13]. For example, Semple et al. [14] conducted an open trial, pre–posttest design study where seven and eight year old elementary students with high levels of anxiety received six weekly, forty-five minutes MBI training using an adapted version of the adult Mindfulness-Based Cognitive Therapy (MBCT) and Mindfulness-Based Stress Reduction (MBSR) intervention programs. The intervention was implemented by a researcher in a small group setting outside of the students’ regular classroom. The finding indicated that the mindfulness training improved students’ anxiety and inattention. However, the sample size was extremely small (five students) and students received the intervention outside of their classroom setting. A recent study using a pretest and posttest pilot quasi-random assignment on 45 students between seven and nine years old was conducted by Nadler et al. [15]. The researchers implemented a 10-min mindfulness practice (mindful stretches with a guided breath-based meditation) or a quiet play activity led by outside instructors and found that students’ self-reported calmness improved. Again, the sample was small, and the intervention was conducted by outside instructors outside the regular classroom. Finally, a curriculum embedded mindfulness study using the Mindfulness Language intervention was conducted by Tarrasch [16] on 242 elementary school students in grades 2, 4, and 6. Students participated in three-months of weekly 45 minutes mindfulness sessions. After the intervention, participants had lower levels of impulsivity, and higher levels of executive attention. This study is a good example of an MBI program implemented in the curriculum, but a 45-min session may not be feasible for many public-school settings with the current fast paced school environment. Therefore, “in the absence of empirically derived guidelines, practitioners should carefully consider evidence for the specific program when selecting from the numerous MBIs available” and his study seeks to address this need [13].

Although previous studies on mindfulness in elementary schools are promising, there is a need for more rigorous evidence-based scientific research in this area. “The use of mindfulness-based interventions (MBIs) in schools has proliferated over the past decade, resulting in the development and marketing of programs and curricula with differences in the underlying content, methods, dosage, and effectiveness” [13]. For example, previous studies have varied in duration and frequency of mindfulness sessions (i.e., dosage) and have been led by external instructors in non-classroom settings. Klingbeil et al. [13] advises that “in the absence of empirically derived guidelines, practitioners should carefully consider evidence for the specific program when selecting from the numerous MBIs available.” Additionally, researchers should be cognizant of developmental theories of younger children in order to avoid “iatrogenic effects that certain practices could have with children of different ages and characteristics” [17]. Researchers need to look at a developmentally appropriate mindfulness program that has specially tailored mindfulness exercises, such as movement and tactile activities.

The purpose of this study is to provide researchers with preliminary findings on the effectiveness of a novel classroom based MBI, called Mind Yeti, for improving the EFs of elementary school students aged 8 to 11 years old. The Mind Yeti curriculum was chosen because it features several administrative components that have been lacking in previous studies: smaller dosage, led by classroom teachers, and delivered in a classroom setting.

## 2. Method

This study is a quasi-experimental design with one group pretest-posttest widely used for prospective and diagnostic purposes across health and educational professional research. The reason for not having a control group was due to the ethical considerations from the school board wanting all groups to participate in the intervention at the same level. In this case an experimental study may simply not be possible [18,19].

### 2.1. Participants

The research protocol and procedures for this study were approved by the Ethics Committee on Animal and Human Experimentation at the Autonomous University of Barcelona (UAB) and were performed in accordance with the relevant guidelines and regulations of the Family Educational Rights and Privacy Act of 1974 (FERPA).

The participants of this intervention were students from a public elementary school in California, USA. The main criterion for choosing this elementary school was convenience: It was available and willing to participate in the research study. Prior to the start of the study, informed consent was obtained from all but two parents. Assent was also obtained from all students. Participants were not financially compensated for their participation in this study.

In light of previous findings that mindfulness training may have a positive effect on elementary students’ behavioral and metacognitive processes [5,20], we decided to make this program available to all students that were enrolled at the public elementary school. Although the entire school participated in the intervention, data was only collected from students aged between 8 and 11 years old in third, fourth, and fifth grades as they were the ones who could best understand and apply metacognitive concepts. In this regard, this was a convenience sample.

Initially, 281 students were selected. However, 104 students were subsequently excluded since they did not complete both the pre- and posttest questionnaires (one third grade and two fourth grade classes). In addition, nine students who incorrectly completed the demographic section (identification login number or grade) of the questionnaire were also excluded from the study.

The final sample (*n* = 177) consisted of 46% female and 54% male. A total of eight classes were included in this study: four third grade classes, one fourth grade class, and four fifth grade classes. Each class had an average of 26 students.

### 2.2. Measures

The Executive Function Student Questionnaire (EFSQ), used to measure the students’ EFs at the beginning and at the end of the study (α = 0.75), was inspired by the Behavior Rating Inventory of Executive Function—Adult Version (BRIEF-A) [21]. (α = 0.73–0.90) and the Executive Function Skills Questionnaire [2]. To develop the EFSQ, the researchers reviewed the questions on the Behavior Rating Inventory of Executive Function [22] and the Executive Function Skills Questionnaire [2]. The questions on the two assessments were compared, and 18 of the most appropriate questions relating to the six EF composites being studied were selected. Some of the questions were adapted to age-appropriate language.

There were 18 questions that covered the six EF subscales, with three questions targeting each EF subscale. Students were asked to answer each item/question by selecting one of the following five options (Likert scale): 1 Strongly Disagree, 2 Disagree, 3 Neutral, 4 Agree, to 5 Strongly Agree. To obtain the students’ self-reported EF composite score for each of the six categories (inhibition, emotional control, sustained attention, working memory, organization, and cognitive flexibility), the mean score of the three questions in each section was calculated. For each EF, the inhibition, emotional control, sustained attention, working memory, organization, and cognitive flexibility, higher mean scores indicated higher levels of EF difficulties. In addition to finding the mean of the categories, the researchers also looked at how students reported on individual items/questions.

Questions were electronically compiled as a questionnaire to be completed online. The morning of the pretest, teachers were sent the link to the EFSQ and then forwarded this link to their students via Google classroom. Students were encouraged by their teachers to answer the questions as best as they could and to ask their teachers for clarification if they had any doubts about the items on the questionnaire. Data collection was administered in the same order in all classrooms and lasted approximately 30 min in each classroom. Due to limited timing and in order to minimize fatigue, students were only required to complete one pretest and one posttest.

### 2.3. Procedure

Previous studies have had sample sizes of less than 100 participants [5,21], with few studies having samples larger than 200 participants [16]. Furthermore, previous studies have implemented MBIs outside of the regular classroom environment [23]. Therefore, in this study, we aimed to expand on previous studies by implementing the intervention in classrooms.

To test our hypothesis, we conducted an intervention where students took part in a mindfulness intervention program using the Mind Yeti curriculum (as recommended by Semple, Droutman, and Reid [24], we used a low-cost, easy-to-implement program that does not require extensively trained facilitators and minimizes disruption of normal classroom activities). Preceding the intervention, the researchers conducted a 15-min presentation to the classroom teachers to inform them of the study, explain the data collection procedure, and answer any questions. Teachers were also sent a document with additional information about the study and the intervention program. They were given a Mind Yeti Playlist to be used as a guide for the 6-week intervention. The Mind Yeti Playlist consisted of 30 different Mind Yeti lessons to be taught over the course of the six weeks. Each week focused on a different core EF through the introduction of different skills, such as focusing the attention on specific sounds, on counting breaths, pretending to be plants (tree in the city), pretending to be animals (whale talk), pretending to be professional workers (sound scientist), identifying their own feelings, and calming down through belly breathing (diaphragmatic breathing). To strength previously learned skills as well as teach new strategies, the week-by-week structure of the program included a lot of overlapping and repetition of previously mindful activities. For example, each week had deep breathing activities which were taught during the first week as a strategy to improve inhibition.

The study used a repeated-measures design with pretest and posttest questionnaires. Teachers and students were assigned confidential Mind Yeti login numbers on the first day of the study. Teachers distributed the numbers to the students. Each student’s number was available to the researcher, classroom teachers, and the student themselves. Teachers were instructed to remind the students to keep the numbers confidential and to discard them after completing the questionnaires. On the first day of the study, the teachers sent out the pretest questionnaires to their students using Google Classroom, and the students emailed their finished questionnaire back to their teacher. Teachers described the study to their students, guided the students through the questionnaires, answered questions, and made clarifications in age-appropriate language. After the six weeks of intervention, the students completed the posttest questionnaire via the same procedure. To guard against biases due to variability in reading proficiencies, the teacher read the questions aloud, and the students marked their answers by selecting one of the five Likert options.

### 2.4. Intervention

The intervention program used was the Mind Yeti curriculum, a resource from Committee for Children. It was designed to help kids reduce stress, improve focus, and build empathy through an app/website-based mindfulness program. Mind Yeti actively engages and guides the students through short, narrated, meditative scripts for a variety of moods: calm down, focus, get along, reset, create, and go to sleep [25]. The program refers to mindfulness as settling the Hubbubbles. The Hubbubbles are essentially some of the EF dysregulations (the distracting thoughts, feelings, and sensations), that students experience throughout the school day. The guided mindfulness sessions from Mind Yeti help students reduce the Hubbubbles, thus improving the EF by helping to boost students’ focus, calming their bodies, and improving their social-emotional learning (SEL).

The Mind Yeti program was selected for this study because it was appropriate for the target population of the study. For example, the Mind Yeti sessions used language that was suitable for students in a school setting between eight and eleven years old. The short duration of the sessions, 5–7 min, was also consistent with the recommendation of previous empirical studies. The program was app-based and was easily implemented in the regular classroom setting without any formal training to teachers. The Mind Yeti program was designed to be an active, mindfulness-learning experience which could take place online and be conducted in the classroom setting. This app-based program also allowed us to gauge the exact time frame of how long students were being mindful based on the length of the mindfulness sessions. Finally, and most importantly, the skills taught with the Mind Yeti curriculum directly targeted the six different EF areas of inhibition, emotional control, sustained attention, working memory, organization, and cognitive flexibility.

The Mind Yeti sessions reduce stress, improve focus, and build empathy through short, narrated, simple, step-by-step, guided, mindfulness practices. The sessions are actively engaging, including a variety of breathing exercises and full-body scans. The mindfulness sessions were primarily audio–visual, with images of the Hubbubbles floating on the classroom whiteboards. Teachers were instructed to play the videos twice daily, three times per week (a total of six times weekly), logged in to the Mind Yeti website—which was automated after the initial setup—and clicked the Mind Yeti Playlist with the predesigned playlist. All the students viewed the images on the whiteboard attached to the projector as they listened to the guided Mind yeti sessions through the overhead classroom speakers which were also connected to the projector. Additionally, the program was app-based and designed to be an active, mindfulness-learning experience that could take place online and be easily implemented in the regular classroom setting. Since the program had guided meditations, sessions could have been implemented without teachers receiving any formal training. Additionally, the program incorporated learning and similar activities from the Second Step curriculum with which the students were already familiar.

### 2.5. Data Analysis

Statistical analysis was accomplished using the Statistical Package for the Social Sciences (SPSS) version 24. Paired-sample *t*-tests were used to assess the significance of pretest–posttest changes in EF subdomain scores. First, we examined the change from pretest to posttest for the whole group (Grades 3, 4, and 5 combined). Then we examined the changes at grade level (excluding Grade 4). The significance level was set at alpha 0.05 (*p* < 0.05). Cohen’s d: small effect 0.2; medium effect = 0.5; large effect = 0.8.

## 3. Results

**Figure 1:**
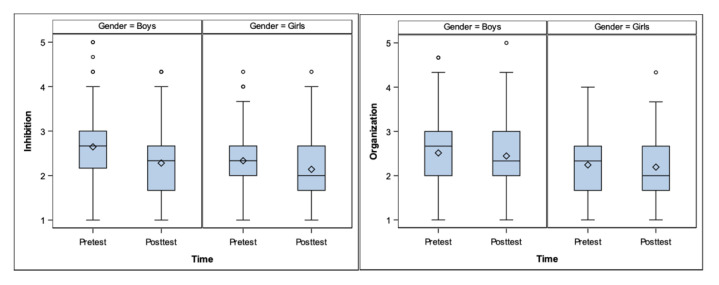
Inhibition and Organization differences according to gender.

For this study, we looked at whether or not there were significant changes from pre- to posttest according to two variables: gender and grade in relation to the EFs. The results showed the following:

Gender: There were significant differences in two of the six EFs. Inhibition (*p* = 0.030) and Organization (*p* = 0.014). See Figure 1. 

**Figure 2:**
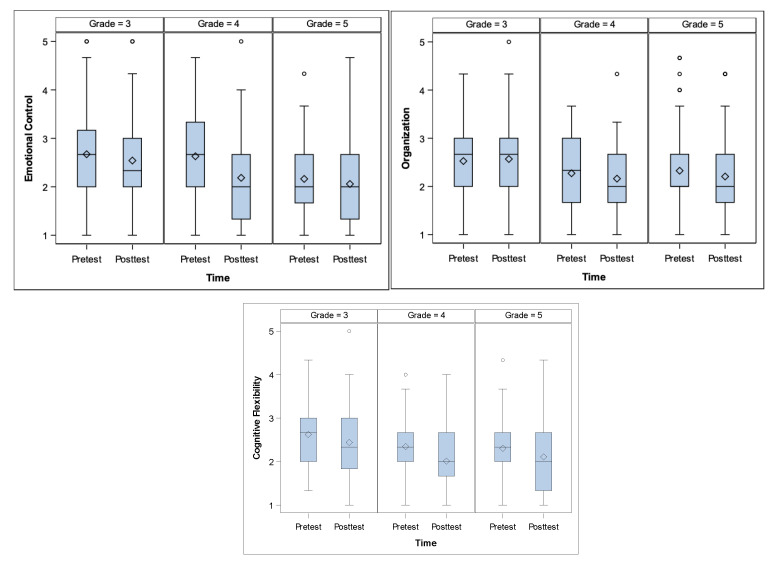
Emotional Control, Organization and Cognitive Flexibility differences according to grade level.

Grade: According to the grade there were significant differences in three of the EFs. Emotional Control (*p* = 0.001), Organization (*p* = 0.028), Cognitive Flexibility (*p* = 0.008). See Figure 2.

Overall, the results from the EFSQ suggested pretest to posttest improvements in students’ three EFs: inhibition (*p* = 0.000), cognitive flexibility (*p =* 0.000) and, working memory (*p* = 0.000). Lower scores reflect higher executive function. Improvement in the EFs is indicated by a decrease in score from pre to posttest. Third grades showed change in two EFs, Inhibition (*p* = 0.100) and cognitive flexibility (*p =* 0.040) whereas fifth grades showed differences in three EFs, Inhibition (*p* = 0.000), cognitive flexibility (*p* = 0.000) and, working memory (*p* = 0.010). The results are summarized in Table 1.

## 4. Discussion

This study was designed to examine the effect of a brief, evidence-based MBI, the Mind Yeti program, on the EFs of elementary school students aged 8 to 11 years old. The aim was to test whether the students’ self-reported scores on each of the EF subscales (inhibition, emotional control, sustained attention, working memory, organization, and cognitive flexibility), significantly changed following the 6-week Mind Yeti program. The hypothesis was that the Mind Yeti program would have a positive impact on all EF subscales.

Results suggested that when elementary students took part in an MBI with specifically tailored administrative components, their levels of inhibition, working memory, and cognitive flexibility were significantly improved from pretest to posttest. The findings partially supported the hypothesis. Previous studies also had inconsistent results, with some studies showing significant improvements [5,20], and others showing non-significant changes [26], in students’ EFs after they participated in an MBI. One similarity between this study and that of Flook et al. [5] is that they also found statistically significant improvements in the areas of Emotional Control (in our study Emotional Control showed differences between males and females, improving among males overtime) and Working Memory. On the other hand, these studies found a statistically significant difference in what they referred to as Plan/Organize (in our study the difference Organization showed differences in terms of gender and grade level, more specifically males performed better in organization and this improvement was also reflected as they grew up comparing fifth with third graders). This could have been due to methodological differences between the studies, especially in the types of mindfulness activities in which the students were engaged in. For example, in the intervention by Flook et al. [5], students engaged in three different types of activities (sitting meditation, games and activities, and modified body scan or meditation), in each session. Whereas, in the present study, the organization activities included imagination and following the breath.

Results were also examined at the grade level. In the area of Inhibition, the findings indicated that at the third and fifth grade levels—as well as for the entire sample, students showed statistically significant improvements in their Inhibition levels, which is in agreement with previous studies. For example, these findings are consistent with those by Felver et al. [20] who conducted a study on the effectiveness of mindfulness to regulate off-task behavior. They found that the Soles of the Feet (SOL) mindfulness intervention may reduce off-task behaviors in third grade elementary school students. They described off-task behavior as “[becoming] angry or defiant,” “refusing to comply with adult directives,” or “becoming frustrated and noncompliant.” Inhibition also showed statically significance in relation to gender, having males scoring higher than females. In the present study, we described these behaviors as examples of not exhibiting inhibition, which is the aptness to resist the temptation to act or change one’s attention, emotions, thoughts, and/or behavior impulsively. In our study inhibition exhibited significance among males having higher scores than females.

Emotional control did not show statically significance as an overall result, but it did show significance in relation to the grade level showing higher scores in fifth grade. The present study is then, consistent with other studies [27,28]. De Carvalho Pinto, and Marôco [27] conducted an MBI using the MindUp curriculum to evaluate its effectiveness on the social–emotional learning (cognitive reappraisal and expressive suppression), of third and fourth grade students. They found a statistically significant difference in expressive suppression (which relates to this study’s emotional control), from pretest to posttest, which partially supports our findings. Riggs et al. [28], investigated the role of meditation on behavior and social–emotional skills in elementary school students. They found that meditation had a positive effect on students’ social–emotional regulation.

Following the Mind Yeti intervention, the present study found a statistically significant improvement in Working Memory scores for the entire sample, and for fifth graders. Similarly, in a pioneering study that investigated the effectiveness of an MBI for working memory capacity (WMC) in adolescents [29], found that, after the intervention, participants showed statistically significant improvements in their WMC compared with the waitlist control groups. The participants in their study were adolescents, somewhat older than the elementary school students in the present study.

In the area of Cognitive Flexibility, following the Mind Yeti intervention, participants in the present study showed a statistically significant improvement for third and fifth grade levels, as well as for the entire sample. This was consistent with the findings of different studies [8,9]. Heeren, Van Broeck, and Philippot [8] conducted a Mindfulness-Based Cognitive Therapy intervention with adults. They found that when adults practiced meditation, they displayed improved cognitive flexibility. Similarly, another study [9] found that mindfulness training may positively influence cognitive flexibility.

Contrary to what was expected, the findings for Sustained Attention, Emotional Control and Organization did not show statistically significant changes from pretest to posttest. Although these results do not support our hypothesis, our findings, particularly for Sustained Attention, are in agreement with those by Tarrasch [16] who conducted a mindfulness intervention on a similar sample. A possible explanation for these results could be that students did not fully understand some of the questions, an argument proposed by Felver et al. [18]. Additionally, as with similar studies [21] students rated themselves very high on the pretest, so there was little opportunity to see growth on the posttest.

Regarding the intervention program itself, Mind Yeti is good for learning how to engage in mindful moments throughout the day and with any mood. However, it is important to consider that some students may require a visual component, which is not part of the current Mind Yeti program. For example, students who are not comfortable closing their eyes, such as those with auditory processing challenges, and even English language learners, may benefit from an additional visual aid on the screen, much like the introductory Mind Yeti video. Additionally, at the time of the intervention, Mind Yeti had not yet published a sequence of lessons. They have since added multiple playlists to their website, classified by grade levels and consisting of 15 sessions each. Nonetheless, Mind Yeti still has the potential to create longer playlists for longer interventions. Since Mind Yeti is relatively inexpensive, and it can be implemented in short dosages with little to no preparation time by the teacher, administrators should consider implementing the Mind Yeti curriculum in their schools.

## 5. Limitations

Despite the positive findings, there were some limitations to the present study. Firstly, there was only one fourth grade class. As we develop clearer theories of the impact of mindfulness intervention for elementary school students, additional participants in the fourth grade group could help further differentiate the key findings of the intervention. Secondly, some classes were discarded from the data since their teachers did not collect both the pretest and posttest data. Specifically, three classes were excluded from the study, and this reduced the sample size and our statistical leverage. This was a particular issue for the fourth grade, where only one teacher collected both pretest and posttest data, meaning that we were unable to compare all three grade levels. A third drawback was that we did not collect longer-term follow-up data. Had we followed students for a period of time after the end of the intervention, this would have allowed us to see how long the effects of the MBI lasted and could aid with planning a cycle of MBI programs, however schools busy daily life makes it very difficult. Finally, the study could benefit from a qualitative analysis to gage students’ perception of the intervention.

## 6. Implication for Future Research

We recommend that future studies consider the limitations of our study and conduct additional studies addressing these limitations. Future research could seek to replicate the findings in our study and to conduct similar studies with even younger elementary school students. There is a need for more classroom-friendly tools to measure the EFs of elementary school students. Additionally, further research should be conducted on how the mindfulness intervention affected the teachers. Since it was not feasible for the present study, researchers should consider conducting randomized studies using the Mind Yeti curriculum. We recommend that Mind Yeti should have a visual component for those who are not comfortable closing their eyes. Students learning English may also find that additional visual aids would assist their engagement. By addressing these limitations, we will be able to deepen our understanding of mindfulness for elementary students, increase the effectiveness of programs for our students, and develop theories from which educators can make more informed decisions.

## 7. Conclusions

This paper makes an important contribution to the literature on the effects of mindfulness training for the EFs of elementary school students. Though our results were mixed, they were also promising. The present study showed that elementary school students’ participation in an MBI was associated with improvements in their level of inhibition, working memory, and cognitive flexibility. These are fundamental skills for academic and social success, and our study demonstrates that they can be enhanced by engaging in mindfulness exercises. At the time of writing, only one study had been conducted using the Mind Yeti curriculum, and that study was conducted on pre-schoolers. To our knowledge, this is the first study conducted to test the effect of the Mind Yeti curriculum on elementary school students. Thus, educators can be more confident that Mind Yeti is an evidence based MBI for use in elementary classrooms.

## Figures and Tables

**Table 1:** Change in executive function (EF) scores from pretest to posttest for the entire sample, 3rd grade, and 5th grade.

	Pretest		Posttest		95% for Mean Difference					
*EFs*	M	SD	M	SD			t	df	*p* Value	Cohen’s *d*
Whole Sample (n = 177)										
Inhibition	2.50	0.77	2.23	0.77	0.19	0.38	5.77	176	0.000 *	0.40
Emotional Control	2.42	0.87	2.25	0.93	0.06	0.27	3.19	176	0.084	0.20
Cognitive Flexibility	2.43	0.69	2.21	0.83	0.12	0.30	4.56	176	0.000 *	0.30
Sustained Attention	3.80	0.63	3.74	0.80	−0.04	0.16	1.16	176	0.247	0.10
Working Memory	2.31	0.85	2.13	0.83	0.09	0.29	3.60	176	0.000 *	0.30
Organization	2.39	0.81	2.33	0.80	−0.05	0.17	1.07	176	0.290	0.10
3rd Grade (n = 64)										
Inhibition	2.58	0.83	2.31	0.81	0.06	0.47	2.55	63	0.010 *	0.32
Emotional Control	2.67	0.87	2.54	0.93	−0.06	0.31	1.39	63	0.170	0.14
Cognitive Flexibility	2.63	0.71	2.44	0.86	0.01	0.36	2.10	63	0.040 *	0.23
Sustained Attention	3.80	0.93	3.74	0.93	−0.054	0.26	1.31	63	0.195	0.06
Working Memory	2.39	0.84	2.35	0.87	−0.14	0.21	0.42	63	0.680	0.04
Organization	2.53	0.83	2.57	0.77	−0.26	0.18	−0.37	63	0.710	0.05
5th Grade (n = 86)										
Inhibition	2.43	0.71	2.14	0.75	0.17	0.42	4.85	85	0.000 *	0.40
Emotional Control	2.16	0.72	2.06	0.86	−0.04	0.25	1.48	85	0.140	0.13
Cognitive Flexibility	2.31	0.65	2.11	0.79	0.17	0.42	4.85	85	0.000 *	0.27
Sustained Attention	3.86	0.69	3.88	0.83	−0.19	0.15	−0.23	85	0.82	0.03
Working Memory	2.19	0.83	1.98	0.76	0.06	0.35	2.79	85	0.010 *	0.26
Organization	2.33	0.82	2.21	0.80	−0.02	0.26	1.70	85	0.090	0.15

* Indicates significant variable.
